# Raft Formation by the Red Imported Fire Ant, *Solenopsis invicta*

**DOI:** 10.1673/031.011.17101

**Published:** 2011-12-19

**Authors:** Benjamin J. Adams, Linda M. Hooper-Bùi, Rachel M. Strecker, Daniel M. O'Brien

**Affiliations:** Department of Entomology, 404 Life Science Building Louisiana State University Baton Rouge, Lousiana 70803

**Keywords:** bubbles, bubble elevator, flood adaptation, plastron

## Abstract

The raft behavior of the invasive red imported fire ant, *Solenopsis invicta* Buren (Hymenoptera: Formicidae), has been documented for over a century. However, no rigorous tests have been performed elucidating the structure, limits, and important characteristics of this behavior. Rafting makes *S*. *invicta* competitive in both native and foreign environments. Further understanding of this behavior will provide critical advancement to the comprehension of this ant's global invasion ecology.

Though speculations exist, no one has looked at the movements of individuals within the raft formation, the longevity of rafts, raft success rate, or the importance of different life stages and varying types of adults to raft formation. Furthermore, bubble use has been extensively studied in arthropods, but it has never been documented in social insects. The use of bubbles as a means of floatation has never before been noted in raft formation.

This study shows that ants trapped under water escape by lifting themselves to the air-water interface through the use of bubbles collected from submerged substrate. The presence of larvae was noted to increase colony survival and maximize raft longevity due in part their ability to hold bubbles under hydrophobic setae.

## Introduction

Originating from a broad floodplain and wetland known as the Pantanal, the red imported fire ant, *Solenopsis invicta* Buren (Hymenoptera: Formicidae) is a polymorphic ant that forms floating conglomerations (referred to herein as rafts) made entirely of intertwined workers, reproductives, and brood (Wheeler 1910; Green *et al*. 1960; Morrill 1974; Jouvenaz 1977; Banks *et al*. 1981; Vinson *et al*. 1986; Anderson *et al*. 2002; Haight 2006; Tschinkel 2006; Chen 2007). This structure is a key contributor to its invasive ability, yet has received little attention by rigorous scientific experimentation with the exception of using this behavior to remove ants from soil (Banks *et al*. 1987).

Outside of South America, the red imported fire ant is highly invasive (occupying > 128 million hectares in the United States alone). It also is established on numerous Caribbean islands, New Zealand, Australia, Taiwan, southern China, and the northern border of Mexico (Sánchez-Peña *et al*. 2005, Tschinkel 2006). Potentially, the ant could invade large areas of coastal Mexico and Central America, the regions immediately surrounding the Mediterranean, the southwestern coast of France, south Britain, southern Japan, southern South Korea, most of Africa, the Middle East, and India (Morrison *et al*. 2004). This ant brings with it large economic costs, exceeding $6 billion a year in the United States (Pereira 2004). From an ecological perspective, *S*. *invicta* lowers native invertebrate and vertebrate diversity and at the same time increases the non-native invertebrate populations (Wojcik *et al*. 2001). The red imported fire ant also shows remarkable plasticity in its biology with mound densities increasing from 30 mounds/ha in native habitats to up to 5000 mounds/ha in invaded environments (Porter *et al*. 1992; Nattrass *et al*. 2001; Tschinkel 2006). In spite of these economic and ecological impacts, the raft behavior of this ant remains largely an uninvestigated phenomenon of social behavior.

Rafting occurs when colonies of red imported fire ants are forced to evacuate their soil nests in order to avoid inundation (Wheeler 1910; Green *et al*. 1960; Morrill 1974; Jouvenaz 1977; Banks *et al*. 1981; Anderson *et al*. 2002; Haight 2006; Tschinkel 2006; Chen 2007). Many researchers describe the central position of the brood (eggs, larvae, and pupae) and reproductives (alates — males, females, and dealated females) in the raft, and state that they are kept out of rising waters by a floating conglomeration (up to 45 cm in diameter) of interconnected worker ants (Wheeler 1910; Morrill 1974; Anderson *et al*. 2002; Haight 2006). Individuals within the central mass are reported to rotate positions, allowing each worker to remain out of the water long enough to prevent drowning (Wheeler 1910). Though no direct studies of longevity have been recorded, these floating masses are suspected to persist for weeks (Morrill 1974; Tschinkel 2006).

In order to establish base-line data for the formation, structure, and longevity of the raft behavior, several series of experiments were performed in which colonies were inundated in order to incite rafting. Our goals were to conduct rigorous observations of both individual and group behaviors of the ants and brood prior to and during raft formation. Furthermore, we aimed to quantify the ability of *S*. *invicta* to maintain the raft structure over prolonged periods of time, ascertaining a maximum time frame of raft longevity.

## Materials and Methods

To study rafting behavior, colonies were collected from fields near Louisiana State University. Collections were made throughout the year in order to eliminate bias due to seasonality. Whole colonies were excavated and placed in painter's buckets (5 gallon, 18.9 liter) with the sides coated in Teflon and brushed with talcum powder to prevent escape. Colonies were chosen on the basis of size, but limited to those nests that would not exceed the volume of the bucket. Time, date, weather conditions, and location characteristics were recorded as well as any observations of brood or reproductives.

Twenty-four hours after collection, colonies were extracted from the soil through preestablished techniques (Jouvenaz 1977; Banks *et al*. 1981; Chen 2007) of slowly adding water to the buckets. Banks *et al*. (1981) techniques were modified by using irrigation tubing with a single drip nozzle instead of the medical intravenous fluid-drip tubing. Water drip rate was monitored once every 30 minutes for the first three hours and recorded to ensure that each colony was flooded at the same rate. When the ants and brood emerged from the soil they were quickly collected and moved to a dry plastic arena with sides coated in Teflon and talcum powder. Ants that were removed from the soil were collected prior to the development of raft formations in order to prevent a bias towards colonies that could successfully raft during flood conditions. Once the ants were removed from the soil and all substrate within the bucket had been submerged, the mixture of soil and water was stirred causing any brood trapped under the soil to float to the surface (Chen 2007). This brood was also collected and placed in the dry plastic arena. Presence of brood, stages of brood, amount of brood, and presence of alated and delated reproductive were recorded once all ants were moved to the dry arena.

After the ant colonies were removed from the soil, the ants acclimated to the lab for a minimum of one week prior to the start of any experiments. Large Petri dishes (150mm × 25mm) with moistened Hydro-Stone (United States Gypsum Company, www.usg.com) were provided for harborage. Harborages were glued to the bottom of the arena to prevent their floatation during experiments. Ants were fed a 20% sugar water solution and crickets *ad libitum*.

To begin each experiment, all debris were cleared from the arena (including midden piles and any uneaten food items) so that no external source aided in flotation. Presence of brood, stages of brood, amount of brood, and presence of reproductives were again noted and compared to previous records for each colony. Initially, 1000 ml of water were quickly added to the arena. After the addition of the first 1000 ml of water, the covers of the large Petri dishes were removed.

Subsequently, water was dripped at a continuous rate of 1500 ml per hour for >3h until all solid substrates were submerged. The water was dripped on the opposite side of the arena from the harborages to reduce disturbance of the raft during formation and initial flotation. To prevent spray from the dripping water coming into contact with the Teflon-coated edges, a splash guard was created by using a two-ounce portion cup with the bottom completely removed. The splash guard was situated immediately below the dripping irrigation hose within the arena and glued in place to prevent flotation.

Conglomerations of ants were considered rafts when multiple layers of workers were observed within the structure and the majority of individuals were free floating on the water with no attachment to solid substrate. During trials, colonies were not allowed to escape from the water. Any colony that successfully escaped the enclosure was removed from the data set. Rafts not maintained for ≥ 12h were considered a failed raft.

Two trials were performed to test the movement (cycling) of ants within the raft. Prior to the formation of the raft structure, but after the addition of the initial 1000 ml of water to the arena, the gasters of 20 major workers (pre-rafting, PR) were marked using modeling paint (Testors Pactra Racing Finish, www.testors.com). Majors were used due to large size and ease of marking. Once the raft was completely formed and no longer connected to any substrate, an additional 20 major workers (post-rafting moving, RM) were marked using a different color of paint. These majors were chosen on the basis of their being on the raft but not within the raft structure (i.e. the ants were part of the floating conglomeration but were freely walking around on top of the structure and were not linked to other ants). A final 20 major workers (post-rafting stationary, RS) were marked using yet another color of paint. These majors were chosen based on their complete integration into the raft structure. These ants had to be interconnected with other ants within the raft and were not capable of freely moving on top of the structure. The number of marked individuals from each color group was counted every hour for two hours. After two hours, the raft was flipped over and marked individuals on the bottom of the raft were counted. Marked individuals were counted again every hour for a total of four hours. Then, the raft was collected and frozen to be kept as a voucher specimen. If cycling was not occurring, a random distribution of PR majors is expected in the raft while all RS and RM majors are expected to be seen at all times on the top side of the raft formation. Any indication of a change in frequency of RS and RM majors indicates movement of ants in the raft. Furthermore, any RS or RM majors found on the bottom of the raft immediately following the flip would suggest cycling into and out of the water.

Location of dealated reproductives as well as the mode of interconnection between individual ants was observed by quickfreezing rafts after > 12hrs of successful rafting. Quick freezing was performed by removing a raft from the water, immediately placing the raft between two sheets of paper towels, and then placing the towels in a zipping plastic bag (Ziploc, www.ziploc.com) within a -85^°^C freezer. Frozen rafts were then dissected after 24hrs. Three rafts were quick frozen and dissected for study of the structure and connections. When present, the location of dealated and alated reproductives within the formation were noted. The mode of interconnection between individual workers was observed under an Olympus SZX12 dissecting microscope (www.olympus.com). The various modes of interconnection were photographed using a Canon D40 (www.canon.com).

Longevity of raft formation and the importance of various life stages were also examined. Once rafts were formed and no contact with solid substrate was occurring, the start time was recorded for the beginning of longevity tests. When the raft completely flattened out, most ants having lost interconnectedness, and all ants were in contact with the water, the formation was no longer considered a raft and complete colony death generally occurred within less than one hour. At this point a final time was recorded and the total rafting time was determined.

The importance of brood was determined by comparing the maximum and average longevity of rafts with and without brood using a chi-square analysis. Also compared were the longevities of rafts with the entire suite of different instars and life stages of brood versus colonies that had only young larvae (1^st^ and 2^nd^ instars) and eggs.

Ants' use of bubbles was recorded through the observation of the behavior of individual ants caught under water during flooding. Photographs and notes were taken of any unusual behavior and the location of bubbles on submerged workers was recorded. Furthermore, the presence of bubbles on larvae was observed under a microscope by submerging individuals and groups of brood underwater in a large Petri dish (150 mm ×25 mm). Due to the extremely hydrophobic nature of brood, individual brood were glued (Loctite Super Glue, www.loctite.com) on their dorsal side to the bottom of the Petri dish for photographs. Observations were made of both glued and unglued brood to ensure no external effects were caused by the glue. Photographs of submerged brood were taken using a Syncrospcopy Automontage system (www.syncroscopy.com). Morphology of larvae of *S*. *invicta* were compared with those of other ant genera including non-flood adapted *Crematogaster*, *Nylandaria*, and *Tetramorium* and a flood-adapted ant in the genus *Linepithema*. Larvae of the aforementioned ant genera were also submerged and observed to determine if bubbles adhered to their cuticles.

**Figure 1.**
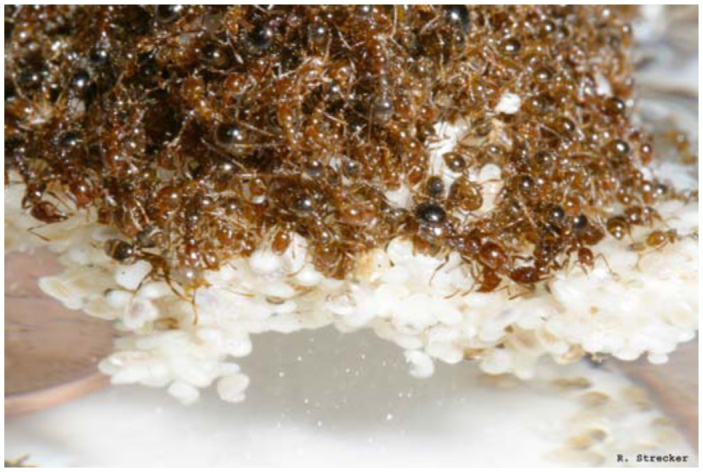
A raft of *Solenopsis invicta* formed by interconnected ants on top of a foundation of brood. High quality figures are available online.

## Results

### Raft formation

When flooded, red imported fire ants migrated out of the soil and aggregated brood at the highest available surfaces. At this point, initiation of the raft formation began with workers interconnecting appendages with one another on top of the brood aggregation. When present, the majority of the brood was used as the foundation of the raft structure with only the smallest of larvae and eggs kept out of the water within the mandibles of adult ants (Figure 1). Before all available substrate submerged, the ants actively tipped the raft into the water and severed the connection with dry land. Workers at the raft's edge splayed out their pro- and mesothoracic limbs onto the water surface, reaching for and connecting to any substrate with which they came in contact. Metathoracic limbs were used to remain interconnected to the raft formation. Several workers and reproductives (often females) never became entwined in the raft structure but instead walked on the top of the interconnected ants. Another notable observation was that on two occasions workers removed all male alates from the raft and pitched them back into the water where they drowned. When present on the raft, male alates were always the first type of individuals to be completely eliminated during prolonged rafting.

### Individual movement

Experiments performed with marked individuals (two trials) showed that the raft is dynamic with interconnected ants constantly cycling. Of the 20 marked ants for each category, fewer were visible with each passing hour. The marked ants quickly dispersed through the raft with less than 25% being visible after the first hour for most groups (PR = 4,5; RM = 3,3; RS = 2,7). After the second hour, less than 10% of the original marked individuals were visible (PR = 2,1; RM =1,1; RS = 1,4). Individuals from both the RM and RS group were found on the bottom indicating a movement of majors from locations above the water to those underneath the water's surface (RM =1,1; RS = 0,1).

### Raft dissections

**Figure 2.**
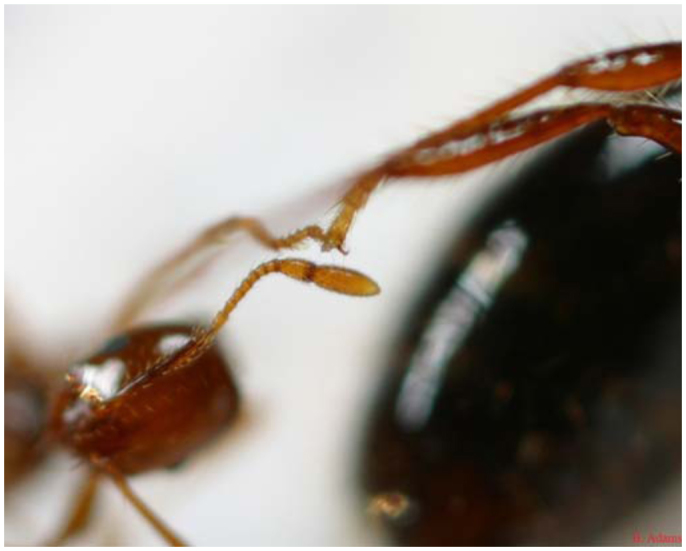
A tarsi-to-tarsi linkage between two *Solenopsis invicta* ants within the raft formation. These ants were separated from a frozen raft. High quality figures are available online.

Dissection of three frozen rafts revealed that interconnected workers link primarily tarsi to tarsi, with occasional connections made tarsi to femur, tarsi to mandibles, and tarsi to propodeum (Figure 2). Once linked together, workers curl in their appendages forming a tight ball of intertwined ants. Dealated females, when present, were found in the middle of rafts. Often male alates, even when present prior to flooding, were not found within the raft structure. Alate females were generally found on the outside of the raft structure. Brood was located on the bottom of the raft except for occasional first instar larvae or eggs which could be found in the mandibles of workers.

### Raft longevity

Colonies capable of successfully creating the raft structure floated from 12 hours to 12 days in undisturbed laboratory trials (n = 11). The presence of brood within the colony was found to increase raft longevity and success rate. Raft structures formed by colonies without larvae failed within 12 hours (n = 4). Red imported fire ant colonies with young larvae (1^st^ and 2^nd^ instars) formed rafts but failed to maintain the raft for longer than one day (n = 2). However, all colonies with 4^th^ instar larvae were capable of rafting for longer periods (n = 5, 3–12 d, 7 ±3.24 d mean ±SD). The success of rafts was significantly affected by the presence of 4^th^ instar larvae (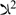
^2^ = 9.09; DF = 3; P = 0.028).No trend existed relating the time of year a colony was collected with its ability to successfully raft or maintain rafts for an extended period 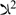
^2^ = 4.41; DF = 5; P = 0.492). The most important characteristic we identified for the formation of successful rafts was the presence of 4^th^ instar larvae.

### Bubble use

Rising waters trapped large numbers of ants and brood in the water several centimeters below the surface. Individual workers that had contact with the substrate walked in a manner similar to the foraging and patrolling behaviors seen on the soil surface under normal conditions (Gordon 1988). As they walked, workers encountered small air bubbles attached to submerged substrate. These bubbles would be actively connected by the workers to their bodies or would passively stick to workers as they encountered them. Other bubbles were also observed trapped on individual ants during submersion. These we termed native bubbles. The passive and active accumulation of bubbles added air to the native bubbles. All bubbles appeared to amass in several locations on the workers' bodies including: behind the head at the connection of the occiput and pronotum, underneath the head capsule, ventrally between the first and second sets of legs near the coxa, directly in contact with the propodeum (generally associated with the propodeal spiracle), and between the postpetiole and the fourth gastral tergite. The ventral bubble and propodeal bubble were the bubbles most often observed increasing in size. Bubbles located on the head and antennae were quickly removed by the ants via grooming behavior. This behavior either removed the bubble entirely from the ant or joined the bubble to the ventral or occiputal surfaces.

Eventually, ants covered with bubbles would lose their footing on the bottom, initiating a rise to the water surface. At this point, the largest ants (major workers) broke the surface tension of the water; minor and median workers, however, often remained caught at the surface but below the air-water interface. When solid surfaces (such as loose grass or the edge of the harborage) that connected the boundary between the water and air were present, median workers were usually able to cross the interface by means of these surfaces. Also, if contact was made with a raft, median workers could escape the surface tension. Unless directly contacted by a raft or other ants, minor workers were usually unable to escape the water and drowned. In the absence of debris to climb on and break the surface tension, the smaller workers usually remained trapped under the water.

**Figure 3.**
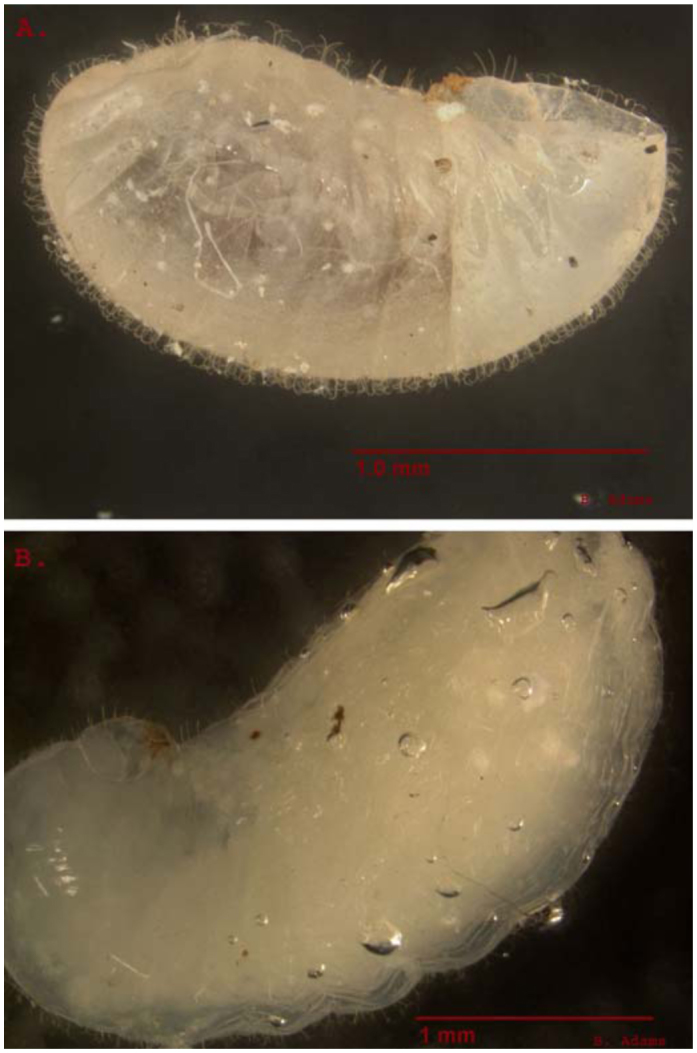
Fourth instar of *Solenopsis invicta*. Setae covering the larvae are forked and recurved towards the body. When submerged these structures assist in holding air bubbles to the surface of the larvae. Notably, setae near the head and buccal pouch of the larvae do not share the forked and recurved characteristics seen on the rest of the body. A is a fourth instar larvae under dry conditions. B is the same larvae submerged under water. High quality figures are available online.

Third and fourth instar red imported fire ant larvae were observed to be densely covered with forked and recurved setae (Figure 3A). When larvae were submerged, bubbles were trapped by these setae against the cuticle — sometimes completely enveloping every part of the larvae except the head, mandibles, and a small area on the ventral surface immediately posterior to the mouth called the praesaepium (Vinson 1997). Notably, setae on the head and within the praesaepium of larvae were neither recurved nor forked.

**Figure 4.**
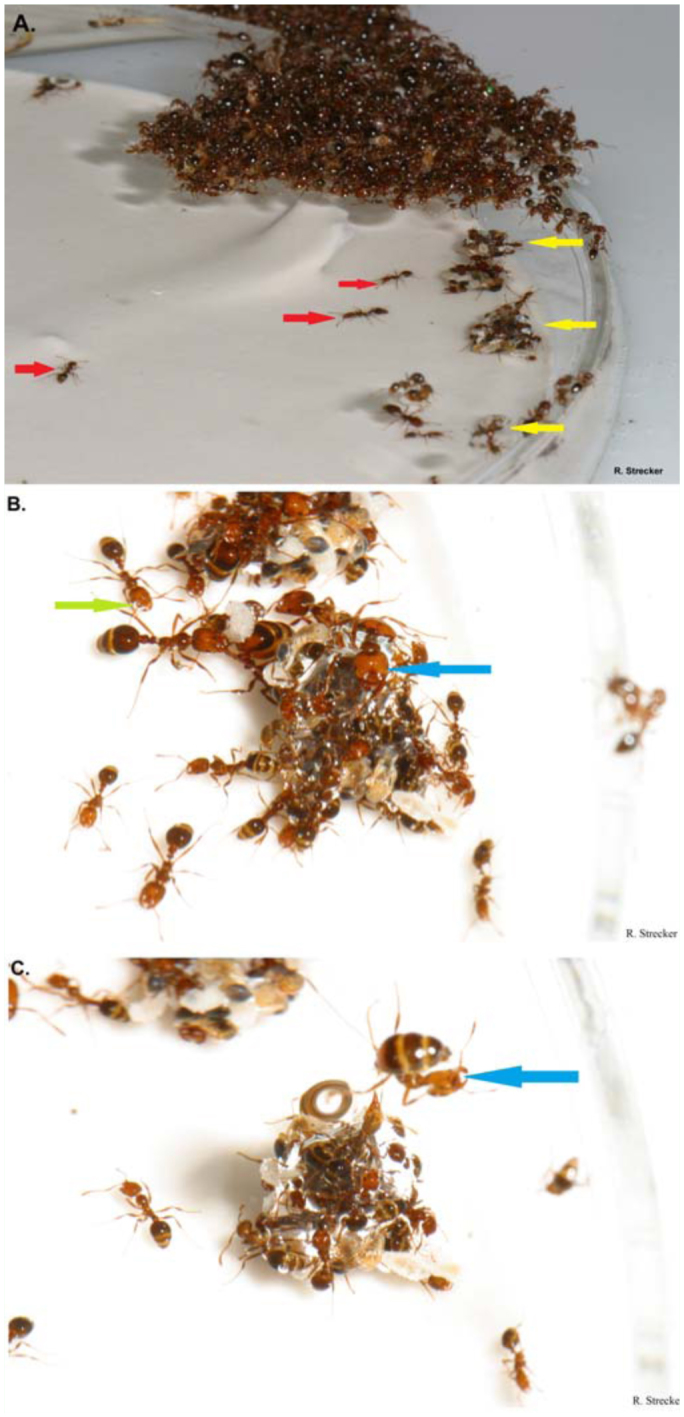
The series of pictures that highlights the behaviors involved in bulloferation. Individual *Solenopsis invicta* ants performing foraging behavior under the water are marked with a red arrow while brood conglomerates are marked with yellow arrows in picture 4A. In picture 4B, a green arrow highlights a bubble being carried by a worker to the brood conglomerations. A blue arrow marks a worker using a rising bubble to escape the water in picture 4B and 4C. All brood conglomerations seen in picture 4A eventually became buoyant and rose to the water's surface. High quality figures are available online.

**Figure 5.**
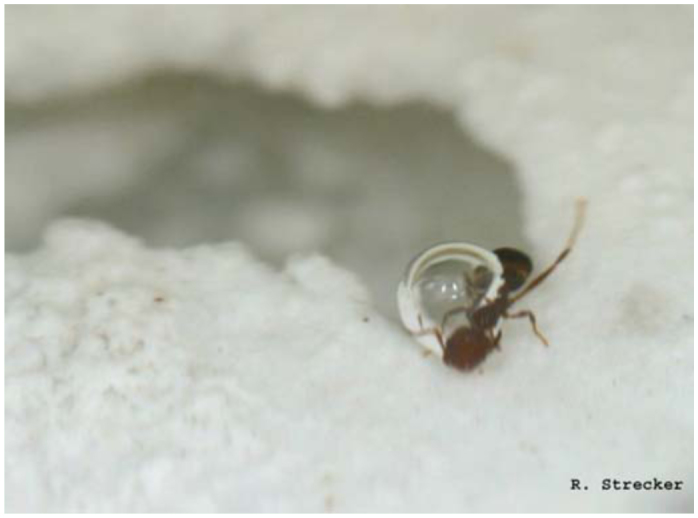
When trapped under the water, *Solenopsis invicta* workers were observed encroaching upon bubbles stuck on submerged substrate. The submerged ants usually contacted bubbles with the locations of their bodies associated with spiracles. High quality figures are available online.

Comparisons of the larvae of *S*. *invicta* with other genera of ants showed variation in setae density and structure. It was observed that *Crematogaster* and *Linepithema* larvae are sparsely covered in setae that are neither forked nor recurved. *Nylandaria* larvae are densely covered with long, straight setae. *Tetramorium* larvae are densely covered in weakly recurved, forked setae. Bubbles were only seen on the cuticular surface of the brood of *S*. *invicta* during submersion whereas the abovementioned species exhibited no bubbles on the surface of the larvae.

Red imported fire ant workers caught under water gathered submerged brood into clusters. Workers then congregated on top of these masses of brood. These brood aggregations were associated with the formation of large bubbles. The bubbles accumulated and joined around the aggregations of brood and workers until buoyancy lifted the entire mass from the submerged substrate. The aggregation would then rise to the water's surface like an elevator, breaking the surface tension with workers in tow (Figure 4).

**Figure 6.**
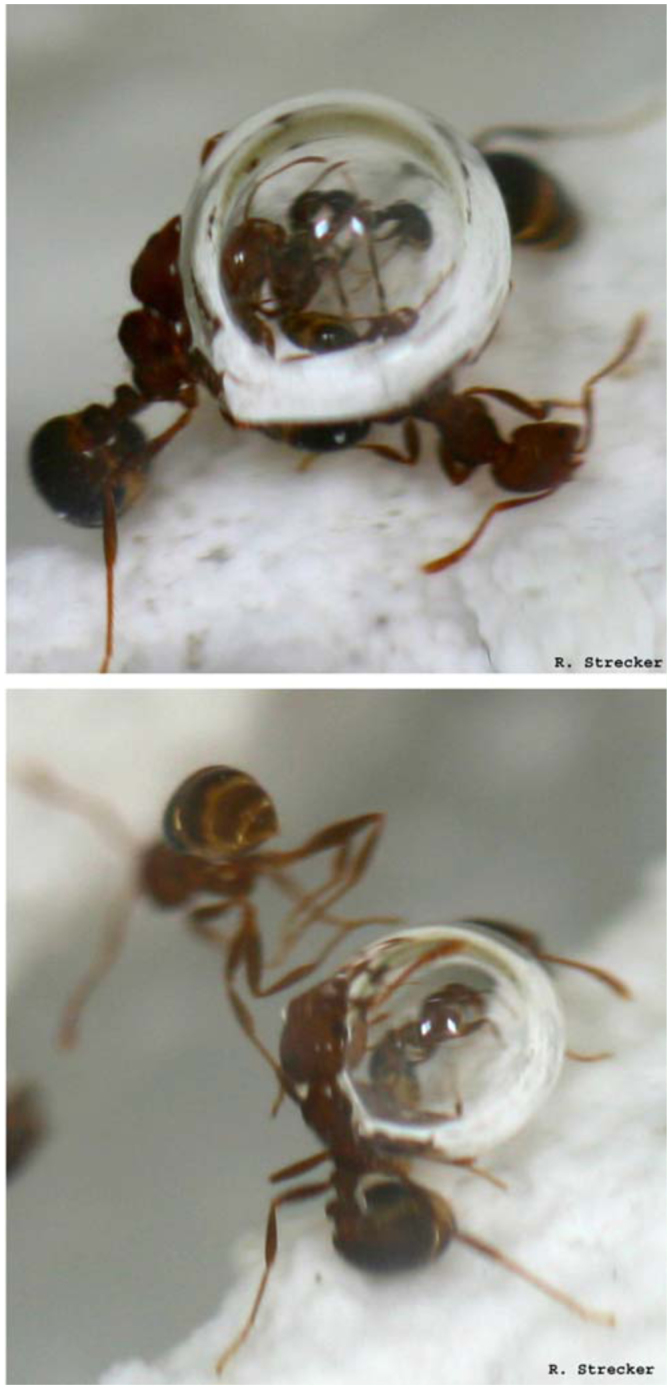
Submerged *Solenopsis invicta* workers use large bubbles to escape the water in the absence of brood. The workers in the second picture immediately rose to the surface on the large substrate bubble when it completely detached from the substrate. High quality figures are available online.

Large bubbles (> 1 mm diameter) trapped on underwater surfaces were often surrounded by submerged worker ants. Workers were observed sidling up to larger bubbles connected to the substrate. Ants allowed the bubbles to contact the areas associated with the spiracles on their bodies (Figure 5). Workers that were gathered around these bubbles were also lifted to the water surface in cases where the bubble detached from the substrate and rose to the air-water interface (Figure 6).

## Discussion

The observations and experiments performed on the formation of the raft structure created by the red imported fire ant, *Solenopsis invicta*, in response to flood conditions support some previous research, indicate a deviation from many previous assumptions, and supply evidence for entirely new behaviors involved in flood avoidance. Support for previous descriptions includes the location of dealated reproductives on the raft, the unimportance of reproductives (male and female) to the immediate raft structure, and the cycling of individual workers within the raft formation (Wheeler 1910; Morrill 1974; Anderson *et al*. 2002). The notable differences from previous descriptions are the location of brood within the raft formation and the overall longevity of the raft (Wheeler 1910; Morrill 1974; Anderson *et al*. 2002; Tschinkel 2006). Several new observations consist of the physical points of connection between individual ants within the structure, the active movement of the raft into rising waters, and the importance of brood and bubbles for successful and prolonged rafting.

No previous methodology or procedures existed directly for the observation of raft formation. The methods for the extraction of colonies of *S*. *invicta* from the soil, however, acted as a starting point (Jouvenaz 1977; Banks *et al*. 1981; Chen 2007). A new addition to the extraction techniques was the initial 1000 ml of water added to the arenas. This addition of water helped to separate any ants that would not normally be a part of the main raft formation in actual flood conditions, including patrollers or foragers. The initial addition of water also helped to consolidate the main portion of the colony in a single area, increasing the likelihood of successful raft formation as indicated by preliminary experiments. Furthermore, the covers of the Petri dish harborages were removed to allow the ants sufficient room to escape the rising water without being trapped. In preliminary experiments, when the tops of the harborages were not removed, rafting failed to occur and most ants drowned within the harborage.

Changes in the presence of marked workers support the observations of Von Ihering quoted in Wheeler (1910). Cycling of workers into and out of the water was confirmed by disappearance of most of the marked individuals from the top of the raft. Cycling was also supported by the presence of both the stationary (RS) and moving (RM) marked majors immediately after flipping the raft. Had no cycling occurred, all 40 of these ants would have been expected to be found on the top of the raft structure and never found on the bottom of the raft structure.

Rafts found without reproductives were first reported by Morrill (1974) and again in Anderson (2002). These statements were confirmed during the present observations of raft formation. The creation of successful rafts without the presence of reproductives is indicative of a worker-mediated catalyst. The elimination of male reproductives from rafts (in many cases by workers removing them), and the persistence of female reproductives during long term inundation indicate a proclivity for the protection of female reproductives during flood conditions.

Dealated females' locations within the center of rafts allow them to persist after long-term flooding. This position provides protection from the potential predation associated with exposure on open water due in part to the increased defensiveness of rafting colonies (Haight 2006). Furthermore, the extremely hydrophobic nature of brood and the centralized location of dealated females suggest that water exposure for dealates probably does not occur. As a result, the lethal fungal infections reported by Morrill (1974) for queens collected immediately after nuptial flights may be reduced during whole colony flotation.

Another new observation is the exact connection points between individuals within the raft structure. The linkage between individual ants and subsequent curling of appendages produced Velcro-like connections in the structure which allowed the raft to be lifted out of the water without breaking the formation. Due to this interlocking, it takes several minutes for the ants to completely disassemble.

A new behavior observed was the tipping of the raft into the water before the connection with solid substrate was severed. This serves as the first documentation of the active movement of the raft formation into the water. Concerted severance effort may occur when the larvae are in the proper position under the raft and to ensure the success of the raft.

Brood location within the raft in these experiments greatly differed from all previous observations (Wheeler 1910; Morrill 1974; Anderson *et al*. 2002; Tschinkel 2006). The location becomes increasingly important when coupled with the new observations of the brood's extremely hydrophobic nature and its tendency to amass bubbles when submerged.

Prior observations may overestimate raft longevity and success. Rafts were previously believed to persist for weeks (14+ days) due to an observation made by Morrill (1974) and later credence given by Tschinkel (2006). Instead, rafts were found to last a maximum of only 12 days in the laboratory with an average longevity of 7 d ±3.24 d and this only occurred when fourth instar larvae were present and abundant. These extended time periods lend support to the importance of the cycling of individuals. In these studies, floating debris and structures exposed through the water were removed and those items did not provide support for rafts, and this may have decreased raft time. However, laboratory flooding trials with soil and floating debris exhibited similar raft longevity (Papillion and Hooper-Bùi unpublished data). Furthermore, it is likely that rafts would not last as long as our trials indicate in nature due to perturbations such as wind and currents.

No success rates have been previously suggested for raft formation. In this work, successful rafts were only formed 64% of the time. The expected longevity and success rate of raft formations is important for predicting red imported fire ant populations after floods subside. Also, a more comprehensive knowledge of the rafting behavior of *Solenopsis invicta* may help to understand and control colony proliferation since it has already been reported that rafting acts as a means of establishing new colonies in previously uninhabited areas (Morrill 1974). In fact, rafting across the Rio Grande is the suspected mechanism for the imported fire ant range expansion into northern Mexico (Sánchez-Peña *et al*. 2005). Finally, these base-line data are necessary for any further observations or experiments more specifically aimed at the suppression of the red imported fire ant via disruption of the rafting ability.

The presence of brood within the colony was necessary for raft formation. In colonies with no larvae, workers produced the raft structure but were unable to escape the surface tension of the water. Cycling individuals effectively sank their nestmates, which resulted in a nearly flat raft within minutes and a failed raft in hours.

The reason for the importance of later life stages of brood became apparent when all four instars were examined under a dissecting scope. Recurved and forked setae completely cover the third and fourth instar larvae. These setae have been previously described as an evolutionary function for allowing the workers to easily stick the larvae together (Vinson 1997; Tschinkel 2006). Submersion of brood under water suggests a different function for the specific shape of fire ant larvae setae. When submerged, these setae trap air bubbles close to the larval cuticle which increases buoyancy. When aggregated into groups, the bubbles surrounding larvae expand presumably from exhalation and deposits of small bubbles made by submerged workers. This behavior acts as a catalyst for a “bubble-powered elevator” that lifts trapped worker ants and brood to the water's surface allowing them to escape drowning.

The most significant new discoveries made were the importance of brood for the survival of individual ants and the formation of the raft structure through the use of bubbles. The use of bubbles by arthropods under flooding or aquatic conditions has been extensively studied, but the use of bubbles as a means of flotation has never been observed in social insects (Chan *et al*. 1972; Elliot *et al*. 1983; de la Torre-Bueno 1989; Decleer 1998, 2003; Perez-Goodwyn 2009; Straton *et al*. 2009). More specifically, the importance of bubbles as an integral part in the behaviors and physical adaptations associated with rafting has not previously been observed in *S*. *invicta*. These trials mark the first observation of bubble use and the possible formation of plastrons and other bubble formations by social insects.

We propose that these newly-discovered behaviors in which ants use bubbles be termed bulloferation. Bulloferation is derived from the Latin words *bulla*, meaning bubble, and *fero*, meaning to carry. This new terminology is important to differentiate the specific repertoire of behavioral uses of bubbles by ants from the previously-coined terms defining particular bubbles in arthropods, i.e. plastrons and compressible air bubbles (de la Torre-Bueno 1989; Straton *et al*. 2009). Specifically, we propose that the use of bubbles as a means to break the surface tension of the water is a newly-documented behavior. Bubbles used in bulloferation are not mutually exclusive from plastrons or compressible air bubbles, but differ due to the ants' complex behaviors associated with them.

The large substrate bubbles may also be of importance in sustaining workers' air supplies long enough to perform bulloferation. The use of substrate bubbles for the purpose of breathing is a newly described behavior and differs from plastrons and compressible air bubbles in that the bubbles are not directly attached to the organism but rather the submerged substrate (de la Torre-Bueno 1989; Straton *et al*. 2009).

The discovery of bubbles and the importance of third and fourth instar brood as vital components of red imported fire ant survival during flood conditions suggest new means of suppression of this invasive ant. Fire ant controls such as insect growth regulators (IGRs) may have a more significant role in fire ant suppression if applied in conjunction with known flooding events such as during hurricane season in the United States or the monsoons in India by reducing or eliminating the later life stages of brood necessary for flood avoidance. Furthermore, soap as a means of sinking rafting colonies may owe its mode of action to removing the bubbles held in place by the hydrophobic setae (Barr *et al*. 1991; Nester 2002).

## **Abbreviations**

PR: pre-rafting
RM: post-rafting moving
RS: post-rafting stationary
